# Comparison of alcalase- and pepsin-treated oilseed protein hydrolysates – Experimental validation of predicted antioxidant, antihypertensive and antidiabetic properties

**DOI:** 10.1016/j.crfs.2021.03.001

**Published:** 2021-03-06

**Authors:** Ruixian Han, Alan J. Hernández Álvarez, Joanne Maycock, Brent S. Murray, Christine Boesch

**Affiliations:** a Nutritional Sciences and Epidemiology, School of Food Science and Nutrition, University of Leeds, LS2 9JT, Leeds, UK; b Food Colloids and Bioprocessing, School of Food Science and Nutrition, University of Leeds, LS2 9JT, Leeds, UK

**Keywords:** Antioxidant, Antidiabetic, Antihypertensive, Angiotensin converting enzyme, Diabetes, Dipeptidyl peptidase IV, α-glucosidase, Hypertension, Oxidative stress, *In silico* prediction, *In vitro* validation, Oilseeds, ACE, angiotensin converting enzyme, DPP-IV, dipeptidyl peptidase IV, E/P, enzyme/protein ratio, FRAP, Ferric reducing power assay, Mw, molecular weight, TEAC, Trolox equivalent antioxidant capability assay

## Abstract

There is emerging evidence on the importance of food-derived bioactive peptides to promote human health. Compared with animal derived proteins, plant proteins, in particular oilseed proteins, are considered as affordable and sustainable sources of bioactive peptides. Based on our previous bioinformatic analysis, five oilseed proteins (flaxseed, rapeseed, sunflower, sesame and soybean) were enzymatically hydrolysed using alcalase and pepsin (pH 1.3 and pH 2.1). Further, low molecular weight (M_w_ ​< ​3 ​kDa) fractions were generated using ultrafiltration. The protein hydrolysates and their low M_w_ fractions were evaluated for their *in vitro* antioxidant, antihypertensive and antidiabetic capabilities, in comparison with samples obtained from two dairy proteins (whey and casein). Apart from dipeptidyl-peptidase IV inhibition, significantly stronger bioactivities were detected for the low M_w_ fractions. In partial agreement with *in silico* predictions, most oilseed hydrolysates exerted comparable angiotensin-converting enzyme inhibitory capability to dairy proteins, whilst whey protein was the most promising source of dipeptidyl-peptidase IV inhibitors. Apart from alcalase-treated soybean, dairy proteins were more efficient in releasing antioxidant peptides as compared to oilseed proteins. On the other hand, soybean protein hydrolysates showed the highest α-glucosidase inhibitory activity amongst all protein sources. Overall, there was limited correlation between *in silico* predictions and *in vitro* experimental results. Nevertheless, our results indicate that oilseed proteins have potential as bioactive peptide sources, and they might therefore be suitable replacers for dairy proteins as well as good sources for development of functional foods.

## Highlights

• Bioactive capabilities of protein hydrolysates show overall limited correlation compared to *in silico* prediction.
• Apart from DPP-IV inhibition, low M_w_ fractions tend to exert more promising biological activities.
• *In vitro* analysis supports oilseed proteins, especially soybean, as good sources of bioactive peptides.
• Confirmation of *in vitro* release of predicted peptides is needed.

## Graphical abstract

**Figure:**
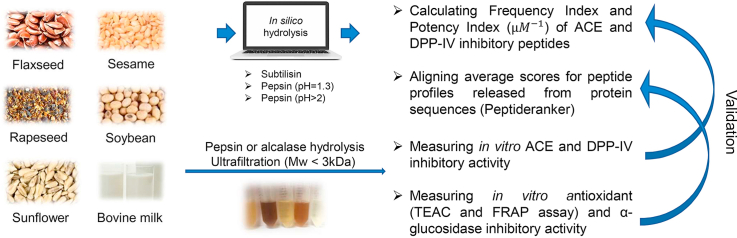

## 1 Introduction

A growing amount of research is focused on developing strategies to valorise food waste and exploit its potential usage for different purposes, including nutrition and health related applications. Proteins from defatted oilseed meal, remainders of oil pressing industries, are extracted from sources such as flaxseed, rapeseed, sunflower, sesame and soybean, and have shown to be promising sources of bioactive peptides with *in vitro* antioxidant (Alashi et al., 2014), antihypertensive (He et al., 2013a), and antidiabetic (Nongonierma and FitzGerald, 2015) properties. Bioactive peptides, defined as peptide fragments of 2–20 amino acid residues in length, are considered to have potential to complement synthetic drugs and become part of new therapeutic strategies against diseases such as hypertension, type 2 diabetes and cardiovascular disease (Nasri, 2017; Patil et al., 2015). There may be some drawbacks, in that plant proteins can be difficult to digest and also some peptides may lead to off flavours, but these negative aspects may depend greatly on the pre-processing/treatment of the protein samples, leading to loss or degradation of unwanted components.

Peptides can be generated from the parental proteins via chemical and enzymatic hydrolysis. Enzymatic methods are preferentially adopted for releasing peptides from precursor proteins because of the specificity of proteases and the mild hydrolysis conditions required that are unlikely to reduce the protein quality and its biological value (Panyam and Kilara, 1996; Tavano, 2013). Nevertheless, protease hydrolysates will contain a wide range of peptides of varying molecular weight (M_w_) and sequences (Sarmadi and Ismail, 2010).

Amongst bioactive peptides, one of the most frequently reported bioactivities refers to antioxidant properties, which can occur via a range of mechanisms, including chelating metal ions, scavenging free radicals and exhibiting reducing power (Elias et al., 2008; Zambrowicz et al., 2015). In addition, angiotensin converting enzyme (ACE) and dipeptidyl-peptidase IV (DPP-IV) inhibitory peptides are well documented as antihypertensive and antidiabetic agents, respectively (Megías et al., 2004; Nongonierma et al., 2017). ACE is a carboxypeptidase, which cleaves a dipeptide (HL) from the C-terminus of angiotensin I, generating angiotensin II, a vasoconstrictor. Meanwhile, this enzyme inhibits and degrades bradykinin, a potent vasodilator (Bénéteau-Burnat and Baudin, 1991). DPP-IV is an enzyme widely recognized for its rapid degradation and cleaving of glucose dependent insulinotropic polypeptide (GIP) and glucagon-like peptide-1 (GLP-1), both incretin hormones being associated with insulin synthesis and secretion (Juillerat-Jeanneret, 2013). Furthermore, bioactive peptides have been reported to suppress postprandial blood glucose via inhibiting α-amylase and α-glucosidase enzymes and potentially attenuate glucose absorption (do Evangelho et al., 2017; Vilcacundo et al., 2017). Both carbohydrase enzymes are critically involved in hydrolysing dietary starch and other long-chain carbohydrates into absorbable monosaccharides (Tundis et al., 2010).

In order to rapidly screen the possible bioactive peptide profiles of proteins, *in silico* approaches have been developed to replace expensive and time-consuming laboratory analyses (FitzGerald et al., 2020). *In silico* analysis is also able to evaluate bioactive potency and thereby allows comparison with other protein sources, such as bovine derived whey and casein, both of which are considered excellent sources of bioactive peptides (Abd El-Salam and El-Shibiny, 2017; Sultan et al., 2018). Although *in silico* prediction may be fast and cost-effective, it is limited by the lack of representative amino acid sequence information in some cases and missing experimental data on the specific enzyme inhibition of all possible peptides, plus a lack of knowledge of activity in real protein mixtures.

In our previous bioinformatics analysis, we screened peptide profiles of a range of proteins generated by *in silico* hydrolysis via subtilisin (alcalase) and pepsin. These results suggested that several oilseed proteins, including napin, cruciferin and glycinin, could generate promising bioactive peptides, especially with ACE inhibitory activity, as compared to dairy proteins (Han et al., 2019). Based on these *in silico* results, it was hypothesized that rapeseed and soybean protein hydrolysates could exert comparable biological activities to those derived from dairy proteins. A key aim of the present study was to validate the *in silico* predictions for ACE and DPP-IV inhibitory activities of oilseed protein hydrolysates through *in vitro* measurements, something that is rarely done. In particular, we evaluated the impact of low M_w_ (M_w_ ​< ​3 ​kDa) peptide fractions *versus* the whole hydrolysates on bioactive properties. In addition, antioxidant and α-glucosidase inhibitory activities were investigated and compared with *in silico* predictions. It should be emphasized that it was important to use recognized methodologies, i.e. we did not aim to develop new analytical tools, although we did adapt the protocols of the enzyme inhibitory assays (ACE, DPP-IV, α-amylase and α-glucosidases), as explained in what follows.

## 2 Materials and methods

### 2.1 Materials and reagents

Pepsin from porcine gastric mucosa, alcalase from *Bacillus licheniformis*, 2,4,6-trinitrobenzenesulfonic acid solution (TNBS), L-leucine, (±)-6-Hydroxy-2,5,7,8-tetramethylchromane-2-carboxylic acid (Trolox), 2,2′-azino-bis(3-ethylbenzo-thiazoline-6-sulfonic acid) diammonium salt (ABTS), potassium peroxodisulfate, sodium acetate-trihydrate, iron (III)-chloride-hexahydrate-solution, 2,4,6-tri(2-pyridyl)-s-triazine, human angiotensin converting enzyme expressed in HEK 293 ​cells, N-[3-(2-Furyl)acryloyl]-Phe-Gly-Gly (FAPGG), captopril, α-amylase from *Aspergillus Oryzae*, starch, α-glucosidase from *Saccharomyces cerevisiae*, *p*-nitrophenyl-α-D-glucopyranoside, human dipeptidyl peptidase IV expressed in baculovirus infected Sf9 cells and diprotin A were purchased from Sigma-Aldrich (Dorset, UK). Acarbose and Gly-Pro *p*-nitroanilide hydrochloride (Gly-Pro-pNA) were obtained from LKT labs (Minnesota, USA) and Cambridge Bioscience (Cambridge, UK), respectively. Oilseeds and dairy proteins were food grade commercial products purchased from local supermarkets (Leeds, UK).

### 2.2 Preparation of oilseed protein isolates

Defatted oilseed meals were prepared using Soxhlet extraction. Briefly, ground oilseeds were mixed with hexane 1:10 (w/v) and the defatted residues recovered after 24 ​h. Residual samples were resuspended in distilled water to a final concentration of 100 ​mg/mL and the pH adjusted to 9.5 with 1 ​M NaOH. After stirring for 4 ​h, the mixture was centrifuged at 3500 x g for 20 ​min and the protein fraction was recovered from the supernatant after adjusting the pH to 4.5 with 1 ​M HCl. Following a second centrifugation, as above, the protein fraction was lyophilized and the protein content of samples was determined using the Kjeldahl method. Different multiplicators were applied for individual proteins (Supplementary Table S1).

### 2.3 Preparation of oilseed and dairy protein hydrolysates and fractions

For pepsin hydrolysis, each protein isolate was suspended in 0.034 ​M NaCl solution with a final protein concentration of 50 ​mg/mL, adjusted to pH 1.3 and 2.1, respectively. Pepsin was added to the protein solution in a 1:25 ​E/P ratio (dry weight of sample x protein content) (w/w) and incubated for 6 ​h ​at 37 ​°C. For alcalase hydrolysis, protein samples were mixed with 0.1 ​M phosphate-buffered saline, pH 8, with an E/P ratio of 1.5:25 (w/w) and incubated 6 ​h ​at 60 ​°C. All enzymatic hydrolysis samples were inactivated by placing them in boiling water for 10 ​min and centrifuged after which the pH of the supernatant was adjusted to 7.0. The degree of hydrolysis (DH) was determined via the trinitro-benzene sulfonic acid (TNBS) method using L-leucine as standard (Adler-Nissen, 1979). In addition, the predicted DH of protein samples was calculated based on the percentage of peptide bonds cut using *in silico* hydrolysis in the whole protein sequence. Low M_w_ fractions of protein hydrolysates were prepared through ultrafiltration using 3 ​kDa molecular weight cut-off membranes (Ultracel® regenerated cellulose, 76 ​mm diameter). Subsequently, samples were lyophilized and stored at -20 ​°C for further measurements.

### 2.4 ACE and DPP-IV inhibitory activity assay

Inhibition of ACE activity was determined according to Vermeirssen et al. (2002) with minor modifications. Briefly, 20 ​μL of sample (1.5 ​mg/mL) was added to 100 ​μL of 1 ​mM FAPGG and preincubated for 10 ​min ​at 37 ​°C. Both sample and substrate were dissolved in 50 ​mM Tris buffer (pH 8.3 with 0.3 ​M NaCl). The reaction was then initiated by adding 20 ​μL of ACE (50 mU/mL in Tris base buffer). The absorbance was recorded over 10 ​min ​at 340 ​nm in 30 ​s intervals using a Tecan Spark10M plate reader. Captopril was used as positive control (IC_50_ ​= ​2.9 ​± ​0.2 ​nM). The DPP-IV inhibition assay was performed according to Nongonierma and FitzGerald (2013) with modifications. Briefly, 25 ​μL of sample (prepared as 1.5 ​mg/mL) was added to 25 ​μL of 10 ​mM Gly-Pro-pNA and pre-incubated for 10 ​min ​at 37 ​°C. The reaction was initiated by adding 50 ​μL of DPP-IV enzyme (500 U/mL). The absorbance was measured at 405 ​nm over 30 ​min in 2 ​min intervals. Diprotin A was used as positive control (IC_50_ ​= ​134.5 ​± ​3.6 ​μM). Inhibition of ACE and DPP-IV were expressed as per cent of non-inhibited control.

### 2.5 Antioxidant activity assays

Antioxidant activity of samples was determined using ABTS radical scavenging (TEAC) and Ferric-Reducing Power Assay (FRAP) assays. ABTS radical stock solution was prepared using 14 ​mM ABTS stock solution and 4.9 ​mM potassium peroxodisulfate and then incubated for 24 ​h in the dark. The ABTS radical working solution was obtained through diluting the stock solution to reach an initial absorbance of 0.700 ​± ​0.020 ​at 734 ​nm. ABTS radical scavenging activity was tested though adding 10 ​μL of sample (1 ​mg/mL, dissolved in distilled water) to 300 ​μL of ABTS radical working solution. The absorbance was taken after 6 ​min ​at 734 ​nm. The FRAP reagent was prepared by mixing 300 ​mM Acetate buffer (pH 3.6), 5 ​mM TPTZ solution and FeCl_3_ in the ratio of 10:1:1 (v/v/v). Reducing capability was measured via mixing 10 ​μL sample (1 ​mg/mL, dissolved in 5% DMSO) with 300 ​μL of FRAP reagent. The absorbance was recorded at 594 ​nm after incubation at 37 ​°C for 15 ​min. Trolox was applied as standard compound in both assays, and antioxidant capability of samples was expressed as mM Trolox equivalents (TE)/g.

### 2.6 α-glucosidase inhibitory activity assay

Inhibitory properties of peptides towards α-glucosidase activity were determined in a microplate based assay according to Zhang et al. (2017) with some modifications. Briefly, 100 ​μL of protein hydrolysate (20 ​mg/mL) was added to 50 ​μL of 0.5 U/mL α-glucosidase solution (dissolved in 0.1 ​M PBS, pH 7.0), and pre-incubated at 37 ​°C for 10 ​min. Then, 50 ​μL of 2.5 ​mM pNPG substrate was added to start the reaction. The absorbance was recorded at 405 ​nm over 10 ​min. Acarbose was used as positive control for α-glucosidase inhibitory (IC_50_ ​= ​1.12 ​± ​0.03 ​mM) assay. Results are expressed in percent of non-inhibited control.

### 2.7 Statistical analysis

**Fig. 1:**
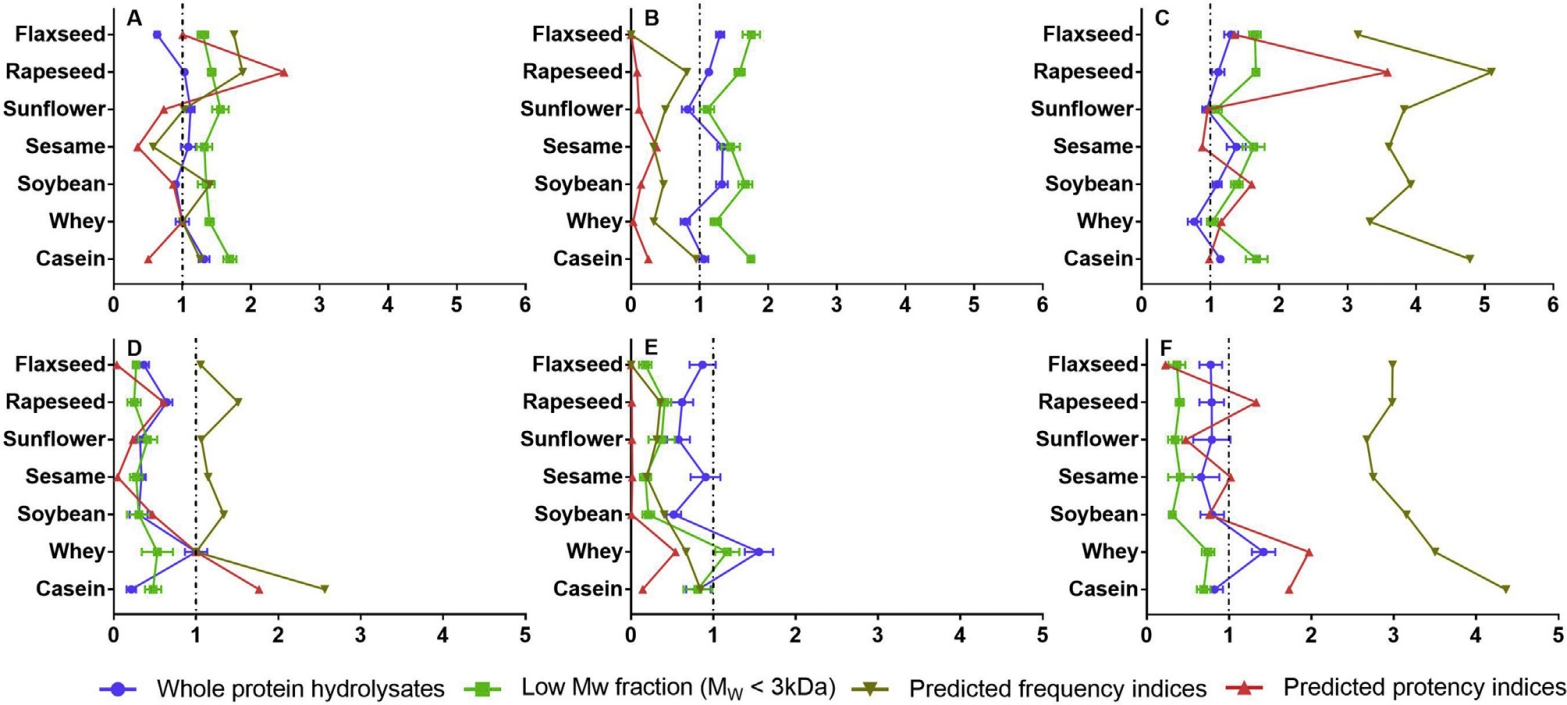
Angiotensin converting enzyme (A,B,C) and dipeptidyl peptidase-IV (D,E,F) inhibitory capability of protein hydrolysates and their low M_w_ fractions obtained using alcalase (A,D), pepsin (pH 1.3) (B,E) and pepsin (pH 2.1) (C,F) hydrolysis, respectively, determined using *in vitro* enzyme assays. Predicted frequency indices and potency indices (mean) are presented to compare with experimental data (mean ​± ​SD).

**Fig. 2:**
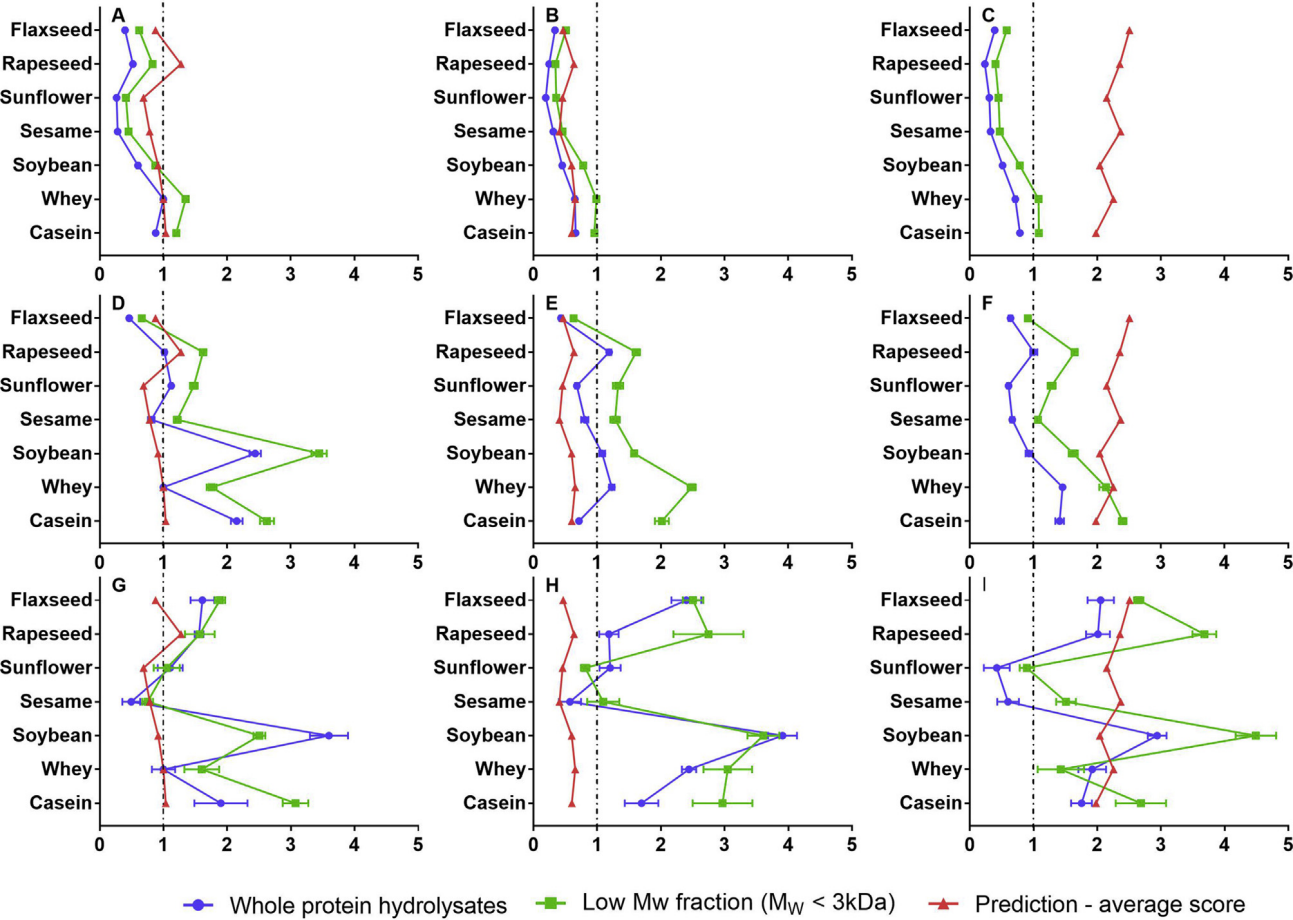
Antioxidant properties determined using TEAC (A,B,C) and FRAP (D,E,F) assays in protein hydrolysates and their low M_w_ fractions obtained using alcalase (A,D,G), pepsin (pH 1.3) (B,E,H) and pepsin (pH 2.1) (C,F,I) hydrolysis, respectively, together with α-glucosidase inhibitory properties (G,H,I) measured using *in vitro* enzyme assay. *In silico* prediction (aligned using PeptideRanker) (mean) was also presented in order to allow comparison with *in vitro* data (mean ​± ​SD).

Detailed results for ACE, DPP-IV, α-glucosidase inhibitory capability together with antioxidant capability of all the protein hydrolysates and their low M_w_ fractions are presented in supplementary Fig. S1, S2, S3, respectively. These single values were then scaled relative to value of alcalase-hydrolysed whey protein. In addition, predicted values were averaged to give a single value for each protein source. Both, experimental results and predicted values are shown in Fig. 1, Fig. 2.

Statistical analysis was performed using the Student’s t-test and two-way analysis of variance (ANOVA) with post hoc analysis (95% confidence interval), depending on the number of groups to compare. Significant differences were considered at p-value < 0.05. Experiments were conducted in triplicate and data were expressed as mean ​± ​standard deviation (SD). The IC_50_ value, defined as the compound concentration inhibiting 50% enzyme activity, was calculated using GraphPad Prism 7.0.

## 3 Results and discussion

### 3.1 Protein content and degree of hydrolysis of protein hydrolysates

The protein contents of oilseed and dairy protein concentrates and isolates varied, ranging from 46.4 ​± ​1.2 to 92.4 ​± ​1.0%, as determined via the Kjeldahl method (Table 1). To take this into account when subsequently assessing the activity of the peptide mixtures, the same concentration of protein was used from each source when conducting protease hydrolysis. The hydrolysis time was 6 ​h; increased DH is not expected if the catalysis time is extended further (do Evangelho et al., 2017; Kimatu et al., 2017).

**Table 1:** Protein contents of protein samples and degree of hydrolysis predicted *in silico* and measured *in vitro.*

Protein	Protein content (%)			Degree of hydrolysis (%)				
		Subtilisin (Alcalase)		Pepsin (pH 1.3)			Pepsin (pH >2)	
		Predicted	*In vitro*	Predicted	*In vitro*		Predicted	*In vitro*
Flaxseed	59.6 ​± ​3.9		23.6 ​± ​0.5^F^		13.1 ​± ​0.8^C^			16.5 ​± ​0.4^E^
Colinin		22.0		8.9			73.8	
Rapeseed	70.3 ​± ​1.5		19.5 ​± ​0.4^E^		9.8 ​± ​1.2^B^			12.2 ​± ​1.2^CD^
Napin		25.1		13.4			70.4	
Cruciferin		29.4		13.8			71.0	
Sunflower	46.4 ​± ​1.2		18.8 ​± ​0.9^E^		9.33 ​± ​0.97^B^			13.3 ​± ​1.6^BD^
11S globulin seed storage protein G3		26.0		12.6			71.3	
2S seed storage protein		16.0		8.5			69.0	
Sesame	92.4 ​± ​1.0		21.7 ​± ​1.7^D^		8.5 ​± ​0.3^AB^			8.4 ​± ​0.8^A^
2S seed storage protein		22.4		8.8			67.3	
11S globulin seed storage protein		27.5		11.6			67.5	
Soybean	75.8 ​± ​0.3		12.4 ​± ​0.7^C^		8.7 ​± ​0.5^AB^			10.7 ​± ​0.8^C^
Glycinin		28.5		11.5			67.2	
Beta-conglycinin, alpha’-chain		26.0		12.7			70.1	
Beta-conglycinin, alpha-chain		26.8		13.9			70.2	
Whey	90.0 ​± ​3.4		25.3 ​± ​1.4^B^		9.2 ​± ​1.5^AB^			12.8 ​± ​2.0^BD^
Beta-lactoglobulin		28.2		17.5			76.3	
Casein	75.8 ​± ​0.3		27.8 ​± ​2.0^A^		7.6 ​± ​1.7^A^			8.2 ​± ​2.0^A^
Beta-casein		33.6		16.1			65.5	
Kappa-casein		29.1		10.6			65.1	

Degree of hydrolysis is expressed as mean with SD of triplicate measurements. Different superscript letters within a column indicate significant differences.

As with our previous bioinformatics analysis, alcalase (pH 8) and pepsin (pH 1.3, pH 2.1) enzymes were utilized to release peptides from the protein samples (Han et al., 2019). Alcalase, a serine S8 endoproteinase family member, has a broad protease specificity with preference for large uncharged residues in P1 position (Adamson and Reynolds, 1996). Pepsin cleavage is more specific at pH 1.3 as compared to pH ​≥ ​2, with a preference to cleave hydrophobic and aromatic residues in the P1 and P1^’^ position (Inouye and Fruton, 1967). Therefore, *in silico* prediction is for DH activity to be in the order of pepsin (pH 2.1) ​> ​alcalase ​> ​pepsin (pH 1.3), yet the TNBS results indicated the highest DH following alcalase hydrolysis amongst all proteins (Table 1). In addition, except for sesame and casein, increasing the pH from 1.3 to 2.1 significantly raised the DH (p ​< ​0.05), however, the value of DH is generally lower than expected. The TNBS assay measures the N-terminal amino groups of proteins, leading to differences in DH calculated based on one or more given protein sequences (Adler-Nissen, 1979). On the other hand, proteolysis is not only ruled by enzyme specificity, but also amino acid profiles, tertiary structure of proteins, minor variations in hydrolysis conditions and sources of protease (Panyam and Kilara, 1996; Tavano, 2013). For example, the folded calyx structure of β-lactoglobulin was reported to be resistant to pepsin digestion, which might explain the much lower DH of whey protein (9.2 ​± ​1.5%) in comparison to calculated DH for β-lactoglobulin (17.5%)

Alcalase exerted the lowest effect with soybean protein (DH 12.4 ​± ​0.7%) (Table 1). It appeared more efficient in liberating peptides from whey (DH 25.3 ​± ​1.4%) and casein (DH 27.0 ​± ​2.0%). Pepsin exerted the most efficient hydrolysis with flaxseed protein, especially at pH 2.1 (DH 16.5 ​± ​0.4%, p ​< ​0.5), while the DH of the other oilseed protein hydrolysates (ranging from 8.4 ​± ​0.9% to 13.3 ​± ​1.6%) was comparable with that of the dairy proteins (ranging from 7.6 ​± ​1.7% to 12.8 ​± ​2.0%). Alcalase and pepsin hydrolysis of whey protein resulted in DH 25.1 ​± ​1.4% and 10.5 ​± ​1.8%, which is within the range reported by Zheng et al. (2008) and Pena-Ramos and Xiong (2001), respectively. Overall, *in vitro* alcalase and pepsin (pH 1.3) treatments resulted in similar DH values to the *in silico* predictions, whilst DH of pepsin (pH 2.1) hydrolysis seemed highly over-estimated in the predictions.

Ultrafiltration methodology was sequentially applied to fractionate the hydrolysed samples to enrich smaller peptides in the M_w_ ​< ​3 ​kDa fractions, which represent the major part of bioactive peptides. Both hydrolysates and < 3 ​kDa fraction were analysed for biological activities.

### 3.2 *In vitro* ACE and DPP-IV inhibitory activity of oilseed and dairy protein hydrolysates

#### 3.2.1 ACE inhibitory activity

Fig. 1 (A,B,C) presents a comparison of ACE-inhibitory activity of the seven protein hydrolysates and their low M_w_ fractions (M_w_ ​< ​3 ​kDa) treated using the three enzyme conditions referred to above, i.e., alcalase, pepsin (pH 1.3) and pepsin (pH 2.1) at the same concentration (1.5 ​mg/mL). All values have been scaled relative to that of alcalase-treated whey protein (46.0 ​± ​4.5%), in order to ease comparison and accommodate the fact that absolute values are subject to variations in protein source and enzyme conditions, as discussed above. Relative values are also advantageous for easier identification of plant proteins that are ‘superior’ to dairy (or other animal-based) proteins. Among alcalase-treated protein samples, the highest ACE inhibitory activity was detected in casein protein hydrolysates (Fig. 1A) followed by rapeseed, sunflower, sesame and whey protein, which all exerted similar inhibitory capabilities (p ​> ​0.05). Soybean presented the second lowest activity, only slightly higher than flaxseed (p ​< ​0.05). With regard to pepsin (pH 1.3) hydrolysis, flaxseed, sesame and soybean exerted similarly high ACE inhibitory properties, stronger than the dairy proteins (Fig. 1B). Apart from soybean, no significant change was found on increasing the pH from 1.3 to 2.1 with pepsin (Fig. 1C). The inhibitory value of pepsin (pH 2.1)-treated soybean protein was lowered to a similar level for the casein, but much more promising than whey protein hydrolysates. Apart from pepsin (pH 1.3)-treated sesame and pepsin (pH 2.1)-treated sunflower protein, the activity of all protein hydrolysates was higher for the low M_w_ (<3 ​kDa) fractions. Similar findings have been reported for tilapia (Raghavan and Kristinsson, 2009) and cowpea (Segura Campos, Chel Guerrero and Betancur Ancona, 2010) protein hydrolysates.

Unlike protein hydrolysates, the low M_w_ fraction of alcalase-treated flaxseed protein exerted a similar activity to rapeseed, sesame, soybean and whey protein hydrolysates. Alcalase-treated casein protein still exerted the most promising inhibition amongst all the low M_w_ samples, but only slightly higher than that of sunflower protein hydrolysates. For pepsin (pH 1.3), casein produced similar activity to flaxseed and soybean protein samples, whilst low M_w_ peptides derived from oilseed exerted a comparable or even higher ACE inhibition compared to those from whey. After the pH of pepsin hydrolysis increased to 2.1, the activity of soybean decreased and consequently the inhibitory capability was significantly lower than casein protein samples, whilst low M_w_ peptides derived from rapeseed protein hydrolysates were now similar in activity with those from casein. Taken together, oilseed proteins should be recommended as potential sources of ACE inhibitors compared to dairy protein, especially whey.

In contradiction to our results, Michelke et al. (2017) demonstrated highest ACE inhibition for whey peptide mixtures, compared with soybean and rice. However, their results were based on tryptophan- and tyrosine-containing dipeptides only, whereas our samples contain a mixture of peptides, and were therefore not limited to dipeptide bioactivity. Interestingly, three ACE inhibitory dipeptides derived from whey protein, IW, WL and VY, were also found in soybean and rice, supporting that plant proteins could be comparable sources for ACE inhibitory peptides (Michelke et al., 2017).

#### 3.2.2 DPP-IV inhibitory activity

Similar to ACE activity, DPP-IV inhibitory activity values, determined at 10 ​mg/mL, were scaled relative to that of alcalase-treated whey protein (43.9 ​± ​6.0%) as summarized in Fig. 1 (D,E,F). Fig. 1D shows that whey protein exhibited the strongest DPP-IV inhibition amongst all alcalase-treated protein samples, followed by rapeseed protein. The other five protein samples exerted similar DPP-IV inhibitory activity (p ​> ​0.05) (Fig. 1E). Similar to ACE inhibition, increasing the pH of pepsin hydrolysis from 1.3 to 2.1 did not increase DPP-IV inhibitory capability. Pepsin-treated whey exerted the most promising DPP-IV inhibitory properties. Pepsin (pH 1.3)-treated soybean showed lower inhibition compared to flaxseed, sesame and casein, but all five oilseed protein samples and casein demonstrated similar inhibitory capabilities after pepsin (pH 2.1) hydrolysis (Fig. 1F).

Turning to the low M_w_ fractions, apart from alcalase-treated casein, the M_w_ ​< ​3 ​kDa peptides did not show higher DPP-IV inhibition than the whole hydrolysates, which is opposite to ACE inhibition. Lacroix and Li-Chan (2012) obtained similar results, reporting inhibition of four fractions to be 63% (<1 ​kDa), 83% (1–3 ​kDa), 82% (3–10 ​kDa) and 78% (>10 ​kDa), respectively. This is in contrast to the work of Konrad et al. (2014), who demonstrated greatest DPP-IV inhibitory properties in peptide fractions below 3 ​kDa, obtained from whey protein hydrolysates using serine protease, and emphasized the highest activity to be in the range of 3–10 ​kDa after further purification. Alcalase-treated whey protein hydrolysates showed relatively lower DPP-IV inhibition, similar to sunflower, sesame, soybean and casein. With regard to pepsin hydrolysates, whey exerted the highest inhibition at pH 1.3 and showed similar inhibition to that for casein at pH 2.1. Flaxseed and sesame were significantly lower than rapeseed (p ​< ​0.05), in contrast to the whole hydrolysates (pepsin pH 1.3), although all five protein samples showed similar activity with pepsin at pH 2.1, which is similar to the trends with the whole hydrolysates. Based on these results, dairy proteins seem to be a better source of DPP-IV compared with oilseeds. In addition, unlike ACE inhibition, whey exerted the highest potential of releasing DPP-IV inhibitory peptides amongst all the proteins. The important active sites and binding sites in ACE and DPP-IV are not identical, which has an impact on the different requirements in terms of amino acid residues, peptide lengths and conformation, which can at least partially explain the different behaviour of whey protein released ACE and DPP-IV inhibitors (Lacroix et al., 2016).

### 3.3 Other biological activities

#### 3.3.1 Antioxidant activity

Despite antioxidant properties of peptides having been widely reported, no specific assay has been developed that quantifies their overall antioxidative potential and summarizes the differing mechanisms of antioxidant and radical scavenging activity (Samaranayaka and Li-Chan, 2011). Therefore, in line with most other work, the present study utilized two established methods to determine antioxidant activity, the TEAC and FRAP assays, which evaluate radical scavenging and metal reducing capability, respectively.

Fig. 2 (A,B,C) shows the results of TEAC in protein hydrolysates and their low Mw fractions. For ease of comparison and in line with other assays, the value for alcalase-treated whey protein hydrolysate, with a TEAC value of 5.48 ​± ​0.12 ​mM ​TE/g, was used to normalize the results. Note that a lower value (1.16 ​± ​0.05 ​mM ​TE/g) was reported by Mann et al. (2015), which might be the result of a lower DH (19.12%). As clearly seen in Fig. 2A, soybean hydrolysates were the strongest antioxidants amongst all alcalase-treated proteins, but still significantly weaker than whey and casein protein hydrolysates (p ​< ​0.05). An increase of ABTS radical scavenging capability was detected amongst most proteins when the pH value increased to 2.1, soybean and sesame protein being the exceptions. In addition, Fig. 2 (A,B,C) clearly showed that, in each case, the low M_w_ fractions showed stronger ABTS radical scavenging activity compared with the whole protein hydrolysates. Foh, Qixing, Amadou, and Xia (2010) and Phongthai, D’Amico, Schoenlechner, Homthawornchoo, and Rawdkuen (2018) also claimed that low M_w_ fractions tended to show better capability of trapping the ABTS radical. Low M_w_ fractions of dairy protein were still the most promising sources of antioxidants, along with their corresponding whole protein hydrolysates. With regard to oilseed proteins, the only difference with the low M_w_ is that the alcalase-treated rapeseed exerted similar capability to soybean. In summary, in alignment with other literature, the TEAC results reflected the superior antioxidant activity of whey and casein compared to oilseed proteins.

The results of the FRAP assay, a frequently applied method to determine antioxidant capability based on electron transfer mechanisms, are displayed in Fig. 2 (D,E,F). Alcalase-treated whey protein only exerted 0.53 ​± ​0.03 ​mM ​TE/g antioxidant capability (used to scale all the other values, as previously), only 9.6% of the value measured via the TEAC assay. Overall, the TEAC and FRAP results seemed weakly correlated (r^2^ ​= ​0.4436), as also shown elsewhere (Choonpicharn et al., 2016; Dong et al., 2013). The reducing power of soybean was the highest amongst all proteins treated with alcalase, while casein exerted the second highest antioxidant activity (p ​< ​0.05) (Fig. 2D). In addition, whey protein also presented mild antioxidant capability after alcalase treatment, stronger than flaxseed and sesame protein hydrolysates. With regard to pepsin (pH 1.3), rapeseed protein hydrolysates exerted comparable antioxidant capability as the whey and soybean protein samples, being more promising than casein and the other oilseed samples. The only decrease of reducing power after raising the pH of pepsin hydrolysis from pH 1.3 to 2.1 was found for the rapeseed and sesame protein (p ​< ​0.05). Both dairy proteins exerted stronger reducing power than oilseeds after pepsin (pH 2.1) hydrolysis. Meanwhile, rapeseed, together with soybean samples, showed the strongest antioxidant activity amongst oilseed proteins.

Comparing the behaviour of whole protein hydrolysates as described above with the low M_w_ fractions, similarly to the TEAC assay, all M_w_ ​< ​3 ​kDa fractions exerted stronger reducing power. In contrast, Arise et al. (2016) reported that only fractions with Mw 5–10 ​kDa from Bambara groundnut protein hydrolysates had promising reducing power, compared to fractions with M_w_ ​< ​5 ​kDa, whereas, He et al. (2013b) showed only fractions with M_w_ ​< ​1 ​kDa exerted measurable reducing power from rapeseed protein hydrolysates. Ajibola, Fashakin, Fagbemi, and Aluko (2011) suggested only fractions with lower M_w_ from African yam bean seed protein hydrolysates were directly linked to stronger reducing activity. As with the whole protein hydrolysate, M_w_ ​< ​3 ​kDa soybean fractions from alcalase-treatment exerted the highest reducing power capability amongst all the protein hydrolysates, 31% and 96% higher than the values for casein and whey protein, respectively. On the other hand, both dairy proteins exerted stronger reducing power capability than soybean and the other oilseed proteins after pepsin (pH 1.3) hydrolysis. Reducing power decreased in sesame and whey protein samples after adjusting pepsin pH to 2.1. The differences amongst pepsin (pH 2.1)-treated low M_w_ fractions were similar with the whole protein hydrolysates. Overall, alcalase-treated soybean protein hydrolysates should be considered as good potential sources of antioxidants, compared with dairy proteins. In addition, whole pepsin (pH 1.3)-treated rapeseed hydrolysates presented noticeably high antioxidant capability, whilst its low M_w_ fraction presented weak reducing power compared to dairy proteins.

#### 3.3.2 α-glucosidase inhibitory activity

Inhibition of α-amylase and α-glucosidase activities are considered as antidiabetic properties. Our samples displayed only very low α-amylase inhibitory activity in whole protein hydrolysates as well as their fractions up to 100 ​mg/mL (raw data not shown). Admassu, Gasmalla, Yang, and Zhao (2018) found that pepsin-treated red seaweed protein hydrolysate exerted 50.3% α-amylase inhibitory activity (1.86 ​mg/mL) and Ngoh and Gan (2016) reported fractions with Mw ​< ​3 ​kDa from protamex treated pinto bean tended to reduce 62.1% of α-amylase activities.

Further investigation was carried out to compare the potential of oilseed and dairy protein releasing α-glucosidase inhibitory peptides at a concentration of 20 ​mg/mL. Fig. 2 (G,H,I) illustrated the α-glucosidase inhibitory capability relative to alcalase-treated whey protein hydrolysates (15.2 ​± ​2.8%) at 20 ​mg/mL. The highest α-glucoside inhibition was found in soybean protein hydrolysates for all three enzyme treatments. Alcalase-treated flaxseed and rapeseed proteins exerted similar α-glucosidase inhibitory capability, compared to casein, but stronger than for the whey protein samples. Using pepsin at pH 1.3, whey protein gave the second strongest inhibition alongside the flaxseed protein hydrolysates. A significant increase of inhibition was detected in rapeseed after raising the pH from 1.3 to 2.1 and consequently it then had similar inhibitory capability to the flaxseed and dairy protein samples. In addition, the sunflower and whey protein hydrolysates showed a slight decrease in inhibitory capability.

After ultrafiltration, the only decrease of inhibitory capability for the low M_w_ fractions was found in alcalase-treated soybean protein, which was then weaker than for casein. The low M_w_ fractions of soybean protein hydrolysates still exerted a noticeable inhibitory capability, the second strongest inhibitor. With regard to pepsin (pH 1.3)-treated hydrolysates, no significant difference was detected in the soybean sample before and after ultrafiltration, but it was still considered as a promising α-glucosidase inhibitor, similar to the whey protein samples. Uraipong and Zhao (2016) reported low M_w_ (<3 ​kDa) fractions from rice bran protein hydrolysates exerted promising α-glucosidase inhibition, while Awosika and Aluko (2019) recommended that high M_w_ (M_w_ 3–5 ​kDa and 5–10 kDa) fractions of yellow field pea protein hydrolysates could exhibit high α-glucosidase inhibition.

A similar tendency of α-glucosidase inhibition was found in the low M_w_ fractions, compared to the whole protein hydrolysates, apart from soybean and rapeseed. Low M_w_ fractions of alcalase-treated soybean exerted weaker inhibition than casein samples. Meanwhile, rapeseed samples were identified as the second promising inhibitors amongst pepsin-treated protein samples. Taken together, soybean protein could therefore be considered as a promising source of α-glucosidase inhibitors, whilst dairy proteins, together with flaxseed and rapeseed, could also be regarded as alternative sources, especially their low M_w_ (<3 ​kDa) fractions.

### 3.4 Comparison with *in silico* predictions

A positive correlation between *in silico* prediction and *in vitro* analysis has been reported in several studies and consequently *in silico* methods have been suggested as a novel and fast screening tool to predict the potential of a protein as a source of targeted bioactive peptides, after hydrolysis by proteases (Gangopadhyay et al., 2016; Hsieh et al., 2016; Wang et al., 2017). In this study, *in vitro* ACE and DPP-IV inhibitory activity of protein hydrolysates were used to validate their predicted frequency index and potency index individually (obtained from the BIOPEP database). The frequency index directly reflects the occurrence of peptides with selected activity (ACE or DPP-IV inhibition) for each protein sequence. Potency indices are a more advanced and accurate parameter because they account for not only the number of the peptides present in a sequence, but also their IC_50_ values (i.e., the concentration of a peptide needed to inhibit 50% of a given bioactivity). Fig. 1. (C, D, F) confirmed this tendency: the potency indices seem closer to the *in vitro* assay values. On the other hand, the *in vitro* antioxidant and α-glucosidase inhibitory data were compared with average scores, which matched closely to those obtained using PeptideRanker, thus referring to the likelihood of released peptides being bioactive. However, overall our work suggested that the frequency indices, potency indices and average scores of fragments of a protein only partly agreed with the *in vitro* experiments. These disagreements are probably mostly due to the incomplete *in vitro* protein hydrolysis and poor representation of the complete range of polypeptides presented in the various sources.

The potential great advantage of *in silico* methods is that they provide a rapid and affordable strategy for predicting and investigating the peptide profiles in proteins. In this approach, peptides released from the precursor proteins are more idealistic, since breakdown of peptide bonds is assumed to occur at very specific cutting sites of the polypeptide chain. However, in real hydrolysis a range of factors including: solution conditions, characteristics of enzyme(s) and substrate(s), protease bioaccessibility (surface activity), presence of protease inhibitors, interactions with other compounds present in the complex food matrix, among others, could lead to incomplete hydrolysis (Amit et al., 2018). Not surprisingly therefore, the predicted DH of the protein sequences were higher than those measured via the *in vitro* TNBS assay, with one exception. This outlier was for flaxseed protein (alcalase and pepsin pH 1.3 treated). Possibly this is explained by the lack of fully sequenced proteins from this source, such as linin - the major storage protein (58–66%) and not available in protein databases, and conlinin 2S (20–42%) the protein used for the *in silico* analysis. In addition, this protein may be more sensitive to protein hydrolysis, thus improving the overall DH obtained experimentally (Rabetafika, H.N. et al., 2011). Of course using the same protease conditions *in vitro* with different proteins from the same plant source can produce different DH, affecting the peptidic profiles of protein hydrolysates (Cheison et al., 2010). Thus some predicted peptides with bioactive properties may not be obtained via *in vitro* hydrolysis due to the disagreement of predicted DH (Chatterjee et al., 2015). The DH of pepsin (pH 2.1) hydrolysed proteins is significantly lower than those predicted by *in silico* analysis, which undoubtedly is the main explanation for the over-estimation of their potential ACE inhibitory capability, DPP-IV inhibitory capability and antioxidant capacity.

Additionally, only a few representative protein sequences were selected for *in silico* analysis, according to their presence in the intact protein sources (Cheung et al., 2009; Gangopadhyay et al., 2016). The plant storage proteins that have their sequences recorded in Uniport database were chosen because these proteins represent a very large proportion of the edible proteins consumed (Shewry and Halford, 2002; Shewry et al., 1995). However, there are other proteins present in these sources that might also release peptides with significant bioactivity.

Also, an absence of standard protocols for enzyme and substrate (protein) preparations, protein hydrolysis and bioassays of bioactive peptides may also complicate the evaluation of the relationship between *in silico* prediction and *in vitro* experiments (Nongonierma and FitzGerald, 2017). Despite this limited correlation, soybean protein was confirmed as a good source for bioactive peptides, especially ACE and α-glucosidase inhibitory peptides. Rapeseed protein is also a good source of α-glucosidase inhibitors, and a notable oilseed protein for releasing antioxidant peptides. However, the moderate levels of ACE inhibition measured experimentally for rapeseed peptides disagreed significantly with the highest predicted frequency and potency indices amongst oilseeds and dairy proteins. This disagreement could be due to the potential interaction of other compounds present in the rapeseed protein hydrolysates (phenolics, carbohydrates, phytates and glucosinolates) that might be interacting or competing with the ACE active site or forming complexes with peptides, thus reducing the ACE inhibition compared to the predicted potency indices (Mansour et al., 1993; Ruan et al., 2021).

Overall, the protein sources with a higher value of frequency index, potency index and/or average scores were supposed to be more likely to release peptides with comparable or more promising bioactive capabilities, in comparison with other sources. The findings are in accordance with the work of (Hsieh et al., 2016) work, who observed a positive correlation between *in silico* and *in vitro* analysis based on this tendency. However, after classifying oilseed and dairy protein sources according to their strength of bioactive capabilities in a descending order, a limited correlation between *in silico* prediction and *in vitro* experiments was detected.

*In silico* tools are, of course, solely based on the protein sequences available in the databases used. They are therefore most reliable and helpful for screening the properties of pure protein samples. The complex structure of biomacromolecules, the interactions between them and with other relevant food components (such as polyphenols) and the food matrix in general, plus the fact that peptides might be produced that are highly bioactive but as yet are not recognized as such, will clearly have a negative impact on the accuracy of *in silico* predictions. Finally, tools such as PeptideRanker are designed for predicting the potential of a peptide to being bioactive, but this is not limited to any specific biological activity, plus these predictions are based purely on structural chemical features (Mooney et al., 2012). It is possible that the high scoring fragments may play roles in biological activities other than those under scrutiny here. In consequence, complete agreement between any experimental assessment and *in silico* analysis is unlikely, unless all the protein sequences are available and their proportions in a protein isolate/concentrate have been clearly identified beforehand, together with the intrinsic and extrinsic factors mentioned above being taken into account.

## 4 Conclusion

In the present study, oilseed and dairy proteins have been demonstrated as good sources of bioactive peptides. Dairy proteins are more promising in releasing antioxidant and DPP-IV inhibitory peptides, while oilseed proteins could be considered as comparable sources of ACE and α-glucosidase inhibitory peptides, especially soybean. Apart from DPP-IV inhibition, ultrafiltration is an approach to enrich targeted bioactive peptides. *In silico* analysis predicted rapeseed and soybean as comparable sources to dairy protein and this was partly born out in the *in vitro* experimental results. However, the relative bioactive capability of oilseeds and dairy proteins predicted by *in silico* and *in vitro* analysis largely disagreed. This disagreement may be largely due to incomplete representation of the full range of protein sequences in the protein isolates/concentrates and/or incomplete enzyme hydrolysis. Nevertheless, this current study provides direct *in vitro* evidence to support the view of replacing dairy proteins with affordable and sustainable oilseed proteins as a source of functional foods, without any apparent drawbacks. Future studies should address the corroboration of the *in vitro* release of peptides and their bioactive properties predicted via *in silico* analysis. In addition, it will be necessary to compare *in vitro* with *in vivo* digestion studies, plus acute or chronic human studies to confirm the predicted health benefits of the peptides released, and its potential to reach the selected target. In addition, the antagonistic and/or synergistic role of polyphenols on the bioactivity of these peptides also needs clarifying.

## CRediT authorship contribution statement

**Ruixian Han:** Conceptualization, Methodology, Investigation, Formal analysis, Writing – original draft. **Alan J. Hernández Álvarez:** Methodology, Writing – review & editing. **Joanne Maycock:** Methodology. **Brent S. Murray:** Conceptualization, Visualization, Writing – review & editing. **Christine Boesch:** Conceptualization, Writing – review & editing, Supervision.

## Declaration of competing interest

The authors declare that they have no known competing financial interests or personal relationships that could have appeared to influence the work reported in this paper.
