# Supplementary material for: Comparison of alcalase- and pepsin-treated oilseed protein hydrolysates – Experimental validation of predicted antioxidant, antihypertensive and antidiabetic properties
Source: Curr Res Food Sci. 2021 Mar 6;4:141–9. doi: 10.1016/j.crfs.2021.03.001 (PMC7985463; doi:10.1016/j.crfs.2021.03.001)
Supplement: Multimedia component 1 [file mmc1.docx]

**Supplementary Table 1**

**A list of nitrogen to protein conversion factor**

| Protein | Conversion factor | Reference |
| --- | --- | --- |
| Flaxseed | 5.41 | ^1^ |
| Rapeseed | 5.6 | ^2^ |
| Sunflower | 5.36 | ^3^ |
| Sesame | 5.30 | Other oilseeds and nuts ^4^ |
| Soybean | 5.5 | An average of soybean ^4^ |
| Whey | 6.15 | Purified milk proteins ^4^ |
| Casein | 6.15 | ^5^ |

**References**

1. B. D. Oomah and G. Mazza, Compositional changes during commercial processing of flaxseed, *Ind. Crops Prod.*, 1998, **9**, 29-37.

2. A. Gorrill, D. Walker and J. Jones, Rapeseed protein sources and amino acid supplementation of diets for weanling rats, *Can. J. Anim. Sci.*, 1974, **54**, 659-667.

3. M. Freer and H. Dove, Rumen degradation of protein in sunflower meal, rapeseed meal and lupin seed placed in nylon bags, *Anim. Feed Sci. Tech.*, 1984, **11**, 87-101.

4. F. Mariotti, D. Tomé and P. P. Mirand, Converting nitrogen into protein—beyond 6.25 and Jones' factors, *Crit. Rve. Food Sci. Nutr.*, 2008, **48**, 177-184.

5. F. W. Sosulski and G. I. Imafidon, Amino acid composition and nitrogen-to-protein conversion factors for animal and plant foods, *J. Agric. Food Chem.*, 1990, **38**, 1351-1356.

**Supplementary figures**

**Fig. 1**

Angiotensin converting enzyme (A,B) and dipeptidyl peptidase-IV (C,D) inhibitory capability of protein hydrolysates (A,C) and their low molecular weight (M_w_ <3 kDa) fraction (B,D) determined using *in vitro* enzyme inhibitory assay. Different capital letters indicate significant differences (p<0.05). * following capital letters represent significant differences between whole protein hydrolysates and their low M_w_ fractions. Error bars represent standard deviations.

**Fig. 2**

Antioxidant activity of protein hydrolysates (A,C) and low molecular weight (M_w_ <3 kDa) fraction (B, D) determined using TEAC (A,B) assay and FRAP assay (C,D). Different capital letters indicate significant differences (p<0.05). * following capital letters represent significant differences between whole protein hydrolysates and their low M_w_ fractions. Error bars represent standard deviation.

**Fig. 3**

α-glucosidase inhibitory activity of protein hydrolysates (A) and low molecular weight (M_w_ <3 kDa) fractions (B) determined using *in vitro* enzyme inhibitory assay. Different capital letters indicate significant differences (p<0.05). * following capital letters represent significant differences between whole protein hydrolysates and their low M_w_ fractions. Error bars represent standard deviation.
